# Activation of a TRP-like channel and intracellular Ca^2+^ dynamics during phospholipase-C-mediated cell death

**DOI:** 10.1242/jcs.152058

**Published:** 2014-09-01

**Authors:** A. Pedro Gonçalves, J. Miguel Cordeiro, João Monteiro, Alberto Muñoz, Paulo Correia-de-Sá, Nick D. Read, Arnaldo Videira

**Affiliations:** 1 IBMC-Instituto de Biologia Molecular e Celular - Universidade do Porto, Rua do Campo Alegre 823, 4150-180 Porto, Portugal; 2 ICBAS-Instituto de Ciências Biomédicas de Abel Salazar, Universidade do Porto, Rua de Jorge Viterbo Ferreira 228, 4050-313 Porto, Portugal; 3 Manchester Fungal Infection Group, Institute of Inflammation and Repair, CTF Building, Grafton Street, University of Manchester, Manchester M13 9NT, UK

**Keywords:** Cell death, Inositol-1,4,5-trisphosphate, Phospholipase C, Staurosporine, Transient receptor potential channel

## Abstract

The model organism *Neurospora crassa* undergoes programmed cell death when exposed to staurosporine. Here, we show that staurosporine causes defined changes in cytosolic free Ca^2+^ ([Ca^2+^]_c_) dynamics and a distinct Ca^2+^ signature that involves Ca^2+^ influx from the external medium and internal Ca^2+^ stores. We investigated the molecular basis of this Ca^2+^ response by using [Ca^2+^]_c_ measurements combined with pharmacological and genetic approaches. Phospholipase C was identified as a pivotal player during cell death, because modulation of the phospholipase C signaling pathway and deletion of PLC-2, which we show to be involved in hyphal development, results in an inability to trigger the characteristic staurosporine-induced Ca^2+^ signature. Using Δ*cch-1*, Δ*fig-1* and Δ*yvc-1* mutants and a range of inhibitors, we show that extracellular Ca^2+^ entry does not occur through the hitherto described high- and low-affinity Ca^2+^ uptake systems, but through the opening of plasma membrane channels with properties resembling the transient receptor potential (TRP) family. Partial blockage of the response to staurosporine after inhibition of a putative inositol-1,4,5-trisphosphate (IP_3_) receptor suggests that Ca^2+^ release from internal stores following IP_3_ formation combines with the extracellular Ca^2+^ influx.

## INTRODUCTION

**Fig. 1.**
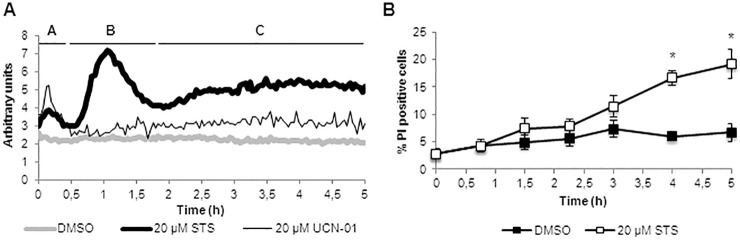
**Staurosporine induces a well-defined Ca^2+^ signature.** (A) Aequorin-expressing wild-type cells grown for 6 hours were incubated with 20 µM staurosporine (STS) or 20 µM UCN-01, and the timecourse emission of luminescence was monitored over 5 hours. The STS-induced Ca^2+^ signature contained two major Ca^2+^ transients (phases ‘A’ and ‘B’) and a third broad [Ca^2+^]_c_ increase (‘C’) and represents an average of 30 independent experiments, each with three to six replicates. The ‘staurosporine-induced amplitude of response’ was calculated by subtracting the solvent DMSO control curve shown in this figure (this was also performed for the following Figs 2–6). [Ca^2+^]_c_ measurements in Figs 1–6 are also presented with errors bars in supplementary material Fig. S1. (B) Cell death as a readout of membrane permeabilization was examined after staining with propidium iodide (PI). Data show the mean±s.e.m.; *P<0.05.

Calcium (Ca^2+^) is an essential intracellular messenger in all organisms, from prokaryotes to humans. The ion binds to a diverse range of proteins, commonly at EF-hand or EF-hand-like domains, and promotes conformational and electrostatic alterations in these proteins. These interactions modulate their activity and contribute to a cascade of signaling events that ultimately defines a Ca^2+^-mediated cellular response to a stimulus (Clapham, 2007). Cells mobilize Ca^2+^ to the cytosol by the opening of channels in the plasma membrane (for Ca^2+^ influx from the external medium) or in the membranes of organelles that act as intracellular stores. In fungi, Ca^2+^ uptake from the extracellular milieu can occur by different mechanisms, depending on the stimulus. The high-affinity Ca^2+^ uptake system (HACS) is the best described fungal Ca^2+^ uptake system. It comprises the channel Cch1 and the regulatory proteins Mid1 and Ecm7 (Martin et al., 2011; Muller et al., 2001). The exact role of Mid1 is unclear, as the protein has also been reported to behave as a non-selective stretch-activated cation channel protein in some systems (Cavinder et al., 2011; Kanzaki et al., 1999; Lew et al., 2008). The HACS seems to be particularly active in minimal medium, whereas the low-affinity Ca^2+^ system (LACS) is more active in nutrient-rich medium (Muller et al., 2001). So far, the only known member of the LACS system is the Fig1 channel, which is involved in mating (Cavinder and Trail, 2012; Muller et al., 2003). A glucose-induced Ca^2+^ influx system was recently proposed (Groppi et al., 2011) but its molecular components are unknown.

In tip-growing organisms such as the fungus *Neurospora crassa*, cytosolic free Ca^2+^ ([Ca^2+^]_c_) is important for polarized growth. Hyphal growth in *N. crassa* has been reported to rely on a tip-high [Ca^2+^]_c_ gradient that is not maintained by extracellular Ca^2+^ influx (Lew, 1999), but is internally derived by means of inositol-1,4,5-trisphosphate (IP_3_)-activated Ca^2+^ channels (Silverman-Gavrila and Lew, 2001; Silverman-Gavrila and Lew, 2002; Silverman-Gavrila and Lew, 2003). Evidence indicates that IP_3_ is generated by a stretch-activated tip-localized phospholipase C that senses tension due to hyphal expansion and converts phosphatidylinositol-4,5-bisphosphate (PIP_2_) to IP_3_ and diacylglycerol (DAG) (Silverman-Gavrila and Lew, 2002; Silverman-Gavrila and Lew, 2003). IP_3_ promotes the release of Ca^2+^ through a large conductance channel, associated with the vacuolar membrane (Cornelius et al., 1989; Silverman-Gavrila and Lew, 2002), and a small conductance channel, associated with endoplasmic reticulum (ER)- and Golgi-derived vesicles that have been proposed to accumulate near the hyphal tip (Silverman-Gavrila and Lew, 2002). Only the latter is believed to be involved in the generation of the tip-high [Ca^2+^]_c_ gradient (Silverman-Gavrila and Lew, 2002; Torralba et al., 2001). However, a recognizable IP_3_ receptor has not yet been identified in fungi (Borkovich et al., 2004; Zelter et al., 2004). The existence of a continuous tip-high [Ca^2+^]_c_ gradient in growing hyphae has recently been challenged (Kim et al., 2012b). In this study, the imaging of [Ca^2+^]_c_, using a genetically encoded Ca^2+^ reporter expressed in *Fusarium* and *Magnaporthe*, demonstrated that [Ca^2+^]_c_ spikes with an irregular frequency occur in growing hyphal tips, with the result that the tip-focused gradient appears and disappears in hyphae extending at constant rates.

The phospholipase C pathway is the main modulator of transient receptor potential (TRP) channels, which are permeable to Ca^2+^. The TRP channel family comprises several subfamilies: classical (TRPC), vanilloid (TRPV), melastatin (TRPM), polycystin (TRPP), mucolipin (TRPML) and ankyrin (TRPA) (Rohacs, 2013). In *N. crassa*, the only known TRP-type channel is YVC-1 (Zelter et al., 2004). In *Saccharomyces cerevisiae*, the homolog of YVC-1 is localized in the vacuolar membrane and is involved in the release of Ca^2+^ into the cytosol (Palmer et al., 2001) after activation by stretch (Su et al., 2009) and PIP_2_ (Dong et al., 2010). Recently, an *in silico* genomic comparison of fungal pathogens identified additional TRP channel homologs (Prole and Taylor, 2012).

The alkaloid staurosporine was initially isolated from *Streptomyces staurosporeus* during a screen for protein kinase C inhibitors (Omura et al., 1977) and was later shown to behave as a broad kinase inhibitor (Karaman et al., 2008). It is largely known to trigger cell death in mammalian models. Although staurosporine exhibits potent anticancer activity, the lack of selectivity and concomitant side effects makes it too toxic for drug therapy. However, the drug remains as an archetypal inducer of cell death and an anticancer agent. Some staurosporine analogs with improved selectivity profiles, such as UCN-01, CGP41251 or PKC412 are currently being evaluated in clinical trials (Gani and Engh, 2010; Gescher, 2000), accentuating the need for understanding the mechanisms of action of this type of drug. We showed recently that *N. crassa* and pathogenic fungi are sensitive to staurosporine (Castro et al., 2010; Fernandes et al., 2013; Fernandes et al., 2011; Goncalves et al., 2014; Gonçalves and Videira, 2014).

In this paper, we have used *N. crassa* cells expressing the codon-optimized, bioluminescent Ca^2+^ reporter aequorin (Binder et al., 2010; Nelson et al., 2004; Troppens et al., 2013) in order to analyze the role of Ca^2+^ signaling during the initiation of fungal cell death by staurosporine. We demonstrate that staurosporine promotes well-defined changes in [Ca^2+^]_c_ with a distinct Ca^2+^ signature and that phospholipase C is a pivotal player during the induction of cell death. The response to staurosporine includes both Ca^2+^ uptake from the extracellular milieu by a novel fungal influx system resembling a TRP channel that seems to be upregulated in the absence of the HACS and an IP_3_-mediated cytosolic recruitment of organelle-stored Ca^2+^.

## RESULTS

### Staurosporine induces a well-defined Ca^2+^ signature

After 6 hours of culture, wild-type *N. crassa* cells expressing the codon-optimized bioluminescent [Ca^2+^]_c_ reporter aequorin (Nelson et al., 2004) were incubated with 20 µM staurosporine, and the luminescence was monitored over time. Staurosporine induced a well-defined signature of [Ca^2+^]_c_ changes (Fig. 1A). The signature included two major Ca^2+^ peaks that we identified as ‘A’ and ‘B’, and a third broad increase in cytosolic Ca^2+^ (‘C’). Peak A occurred immediately upon addition of staurosporine and lasted for ∼20 minutes, and peak B, having the greatest amplitude, occurred after 35–40 minutes and lasted for ∼80 minutes. UCN-01, a natural stereoisomer of 7-hydroxystaurosporine currently in clinical trials for cancer treatment (Gani and Engh, 2010), also provoked an immediate peak of [Ca^2+^]_c_, although the overall Ca^2+^ signature was different from that caused by staurosporine (Fig. 1A).

The levels of staurosporine-induced cell death, as measured by staining with propidium iodide, a reporter of plasma membrane permeabilization, were only significantly increased after 3 hours of incubation with the drug (Fig. 1B; representative images of propidium iodide staining at different time-points are presented in supplementary material Fig. S2A). This supports the view that peaks A and B, that occur within the first 2 hours of treatment with staurosporine, correspond to specific Ca^2+^ signaling events upstream of cell death and do not involve non-specific Ca^2+^ entry into the cytosol due to plasma membrane disruption. The third phase (C) of the staurosporine-induced Ca^2+^ signature coincides with a significant increase in the percentage of propidium-iodide-positive cells (Fig. 1B). Total available aequorin for Ca^2+^ detection was not affected by the drug throughout the entire length of the staurosporine-induced Ca^2+^ signature (supplementary material Fig. S2B).

### The staurosporine-induced Ca^2+^ signature is derived from extracellular and internal Ca^2+^ stores

**Fig. 2.**
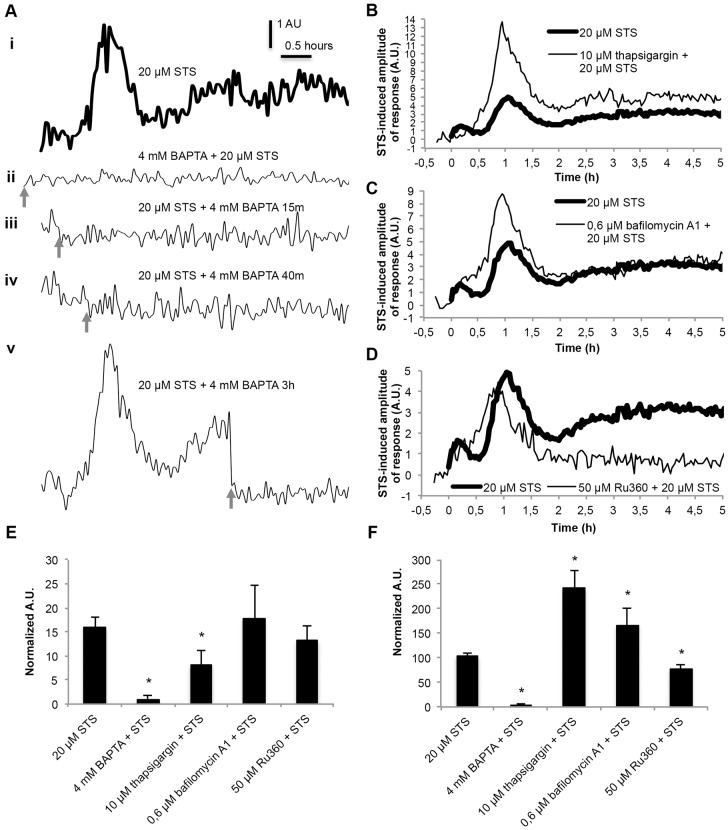
**The staurosporine-induced Ca^2+^ signature results from Ca^2+^ influx into the cytosol from the extracellular medium and from internal Ca^2+^ stores.** (A) The influence of BAPTA on the Ca^2+^ signature after aequorin-expressing wild-type cells were treated with 20 µM staurosporine (STS). (i) No BAPTA control. (ii–v) 4 mM BAPTA was applied at the indicated time-points (arrows). The figures represent the staurosporine-induced amplitude of response. (B–D) The influence of 10 µM thapsigargin (B), 0.6 µM bafilomycin A1 (C) and 50 µM Ru360 (D) on the staurosporine-induced Ca^2+^ signature response in aequorin-expressing wild-type cells. (E,F) Quantification (in arbitrary units, A.U.) of the [Ca^2+^]_c_ transients A and B, respectively, which are shown in A–D. Data show the mean±s.e.m.; *P<0.05.

In *N. crassa*, free Ca^2+^ is stored in different intracellular organelles and in the cell wall, and its distribution depends on the growth phase (Bowman et al., 2011; Naveena Lavanya Latha and Maruthi Mohan, 2011; Torralba et al., 2001). We aimed to identify the source(s) of Ca^2+^ that give rise to the staurosporine-induced [Ca^2+^]_c_ signature, and we started by looking at the contribution of extracellular Ca^2+^. Pre-incubation with the membrane-impermeable Ca^2+^-chelator BAPTA resulted in complete abolition of the [Ca^2+^]_c_ increases associated with the staurosporine-induced Ca^2+^ signature (Fig. 2Ai,ii,E,F). BAPTA added at later time-points after the incubation with staurosporine (15, 40 or 180 minutes) abolished subsequent elevations in [Ca^2+^]_c_ of the Ca^2+^ signature (Fig. 2Aiii–v). This indicates that Ca^2+^ influx occurs continuously throughout the staurosporine-induced Ca^2+^ signature. Because non-specific Ca^2+^ influx does not occur during phases A and B of the Ca^2+^ signature (see previous section), our results point to the presence of Ca^2+^-permeable channels in the plasma membrane that are involved in generating these [Ca^2+^]_c_ transients.

The contribution of the ER to the staurosporine-induced Ca^2+^ signature was evaluated by treating cells with the ER-selective Ca^2+^-ATPase inhibitor thapsigargin. Thapsigargin causes the depletion of ER Ca^2+^ and prevents Ca^2+^ sequestration by this organelle (Thastrup et al., 1990). Pre-treatment with thapsigargin changed the signature of the Ca^2+^ response to staurosporine and resulted in the absence of peak A (Fig. 2B,E,F). Because Ca^2+^ in the ER is likely depleted by thapsigargin before the addition of staurosporine, this result is consistent with Ca^2+^ release from the ER contributing to peak A. However, inhibition of the ER Ca^2+^-ATPase resulted in an increase in the amplitude of peak B compared with that of the untreated control, suggesting that ER Ca^2+^-ATPase activity plays a significant role in sequestering Ca^2+^ during this second phase of [Ca^2+^]_c_ increase. The amplitude of the prolonged [Ca^2+^]_c_ elevation during phase C was also increased in cells that were pre-treated with thapsigargin, indicating that continued Ca^2+^-ATPase activity might also occur during this period.

In *N. crassa*, the vacuoles are responsible for the sequestration of Ca^2+^ under stress conditions to avoid the toxic accumulation of Ca^2+^ in the cytosol (Cornelius and Nakashima, 1987). We used bafilomycin A1 to assess whether the complex and heterogeneous vacuolar system in *N. crassa* (Bowman et al., 2011) plays a role as a Ca^2+^ store during the staurosporine-induced Ca^2+^ signature. Bafilomycin A1 has previously been used to inhibit Ca^2+^ uptake by vacuoles and smaller acidic vesicles by blocking H^+^-ATPase activity, which results in disruption of the proton gradient required for Ca^2+^ uptake by the vacuolar Ca^2+^/H^+^ exchanger (Cordeiro et al., 2013). The presence of bafilomycin A1 resulted in a [Ca^2+^]_c_ increase in peaks A and B but not during peak C following staurosporine treatment (Fig. 2C,E,F). These results are consistent with the vacuolar system playing a role in sequestering Ca^2+^ during phases A and B of the staurosporine-induced Ca^2+^ response but, in contrast to the ER, not during phase C.

Mitochondria are also involved in Ca^2+^ sequestration. In mammalian cells, Ca^2+^ uptake by mitochondria occurs mainly through the mitochondrial Ca^2+^ uniporter (MCU), which is putatively homologous to the *N. crassa* protein NCU08166 (Bick et al., 2012). Pre-incubation with the MCU-specific inhibitor Ru360 (Zazueta et al., 1999) before the addition of staurosporine converted the [Ca^2+^]_c_ peaks associated with phases A and B into a single peak and caused the abolition of the extended increase in [Ca^2+^]_c_ during phase C (Fig. 2D–F). These results suggest that mitochondria play a role in the sequestration of Ca^2+^ during phases A and B. Taken together, our results point to a complex and dynamic response to staurosporine, wherein cells mobilize Ca^2+^ from and to the extracellular medium, ER, vacuoles/acidic organelles and mitochondria.

### Staurosporine activates phospholipase-C- and IP_3_-mediated recruitment of Ca^2+^

Phospholipase C converts PIP_2_ into IP_3_ and DAG. IP_3_ acts as a second messenger and, by binding to its receptor, results in Ca^2+^ mobilization from intracellular stores, whereas DAG activates protein kinase C (PKC) (Berridge, 2009). In *N. crassa*, IP_3_ has been reported to promote the release of Ca^2+^ from the ER (Silverman-Gavrila and Lew, 2002) and vacuole (Cornelius et al., 1989; Silverman-Gavrila and Lew, 2002). Given the importance of phospholipase-C–IP_3_ signaling for hyphal growth (Silverman-Gavrila and Lew, 2001; Silverman-Gavrila and Lew, 2002; Silverman-Gavrila and Lew, 2003), we hypothesized that it could be involved in the fungal response to staurosporine.

**Fig. 3.**
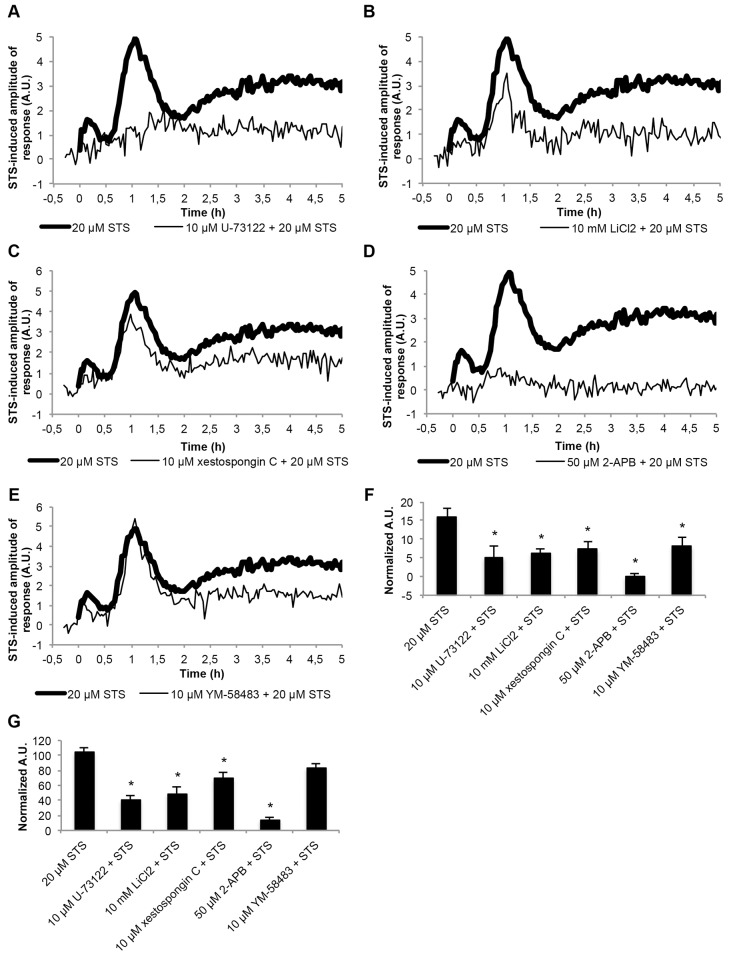
**Staurosporine activates phospholipase-C- and IP_3_-mediated recruitment of Ca^2+^.** (A–E) The influence of pre-treatment with different inhibitors on the Ca^2+^ signature after aequorin-expressing wild-type cells were treated with 20 µM staurosporine (STS). Cells were pre-treated with 10 µM U-73122 (A), 10 mM LiCl_2_ (B), 10 µM xestospongin C (C), 50 µM 2-APB (D) and 10 µM YM-58483 (E). (F,G) Quantification (in arbitrary units, A.U.) of the [Ca^2+^]_c_ transients A and B, respectively, which are shown in A–E. Data show the mean±s.e.m.; *P<0.05.

We measured [Ca^2+^]_c_ dynamics in response to staurosporine treatment in cells pre-treated with the phospholipase-C-selective inhibitor U-73122 (Smith et al., 1990). The [Ca^2+^]_c_ response over the entire 5-hour timecourse was greatly suppressed (Fig. 3A,F,G), consistent with a significant requirement for phospholipase C during this response. Lithium (LiCl_2_) has been used in *N. crassa* to inhibit inositol monophosphatase, thus preventing the synthesis of phosphoinositides (Hanson, 1991). This inhibition reduces phospholipase C activity owing to the absence of adequate levels of PIP_2_ to be hydrolyzed into IP_3_ and DAG (Berridge and Irvine, 1989). LiCl_2_ in the presence of staurosporine significantly reduced the [Ca^2+^]_c_ peaks during phases A and B and almost completely abolished the [Ca^2+^]_c_ increase of phase C (Fig. 3B,F,G). These effects on [Ca^2+^]_c_ were very similar to those resulting from the effects of the IP_3_-receptor-selective inhibitor xestospongin C (Gafni et al., 1997) (Fig. 3C,F,G). This suggests that LiCl_2_ and xestospongin C act by preventing IP_3_ formation and the consequent release of Ca^2+^ from internal stores that seem to participate early on but continue to be involved throughout the entire [Ca^2+^]_c_ response to staurosporine. We also tested the effect of 2-APB, which has been previously shown to block an IP_3_-receptor-like channel and hyphal growth in *N. crassa* (Silverman-Gavrila and Lew, 2002). 2-APB blocked most of the [Ca^2+^]_c_ response to staurosporine, exhibiting a stronger inhibitory effect than xestospongin C (Fig. 3D,F,G). Although 2-APB might be blocking IP_3_-receptor-activated Ca^2+^ release, it is likely that its effects result from the inhibition of TRP channels, as previously claimed (Clapham et al., 2005). This might explain its different effects on the staurosporine-induced Ca^2+^ signature when compared with the more selective IP_3_ receptor inhibitor xestospongin C. Taken together, these results indicate that staurosporine promotes the activity of phospholipase C and that the recruitment of Ca^2+^ from intracellular stores requires the generation of IP_3_.

Because Ca^2+^ influx from the extracellular space can be triggered by the depletion of IP_3_-sensitive Ca^2+^ stores through store-operated Ca^2+^ entry (SOCE, or capacitative Ca^2+^ entry) (Putney, 1986), we evaluated whether SOCE was triggering the Ca^2+^-release-activated Ca^2+^ channel (CRAC). We pre-treated cells with the CRAC-selective inhibitor YM-58483 (Zitt et al., 2004) before incubation with staurosporine, and we observed that the beginning of the [Ca^2+^]_c_ peak A was present, probably corresponding to the IP_3_-activated intracellular store depletion phase, whereas the second part of the same peak was significantly reduced (Fig. 3E–G). Phase C was also decreased in YM-58483-pre-treated cells. Thus, it seems that the staurosporine-induced Ca^2+^ response in *N. crassa* includes the process of SOCE, which has not been reported in filamentous fungi before.

### PLC-2 regulates staurosporine-induced cell death and polarized hyphal growth

**Fig. 4.**
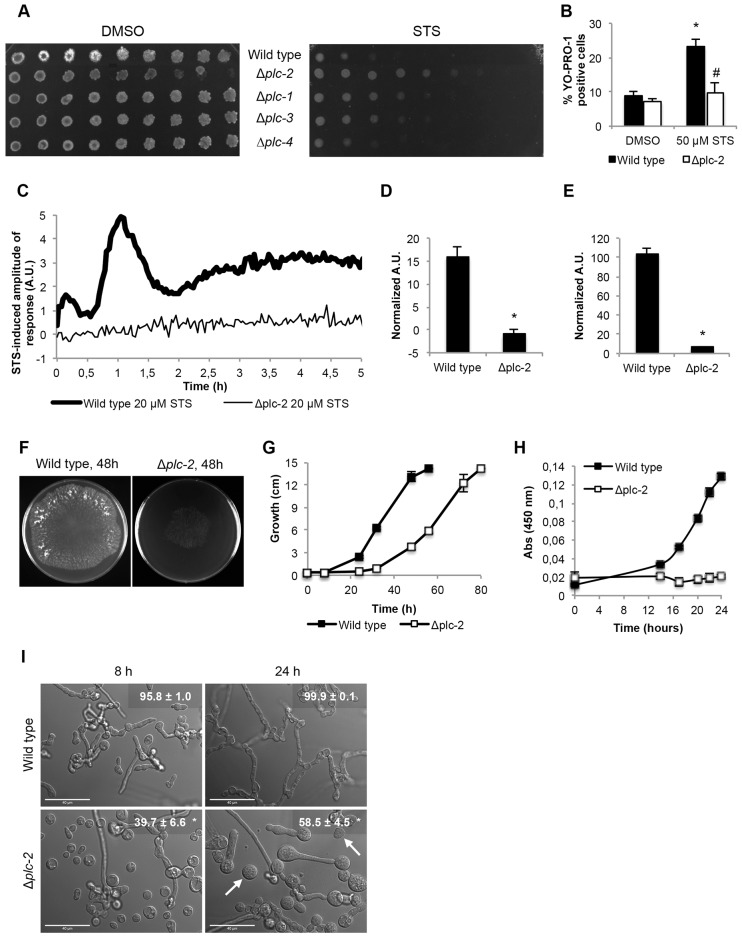
**PLC-2 is required for the Ca^2+^ signature and for cell death induced by staurosporine, and is involved in *N. crassa* hyphal development.** (A) The sensitivity profiles of deletion strains for the four *N. crassa* phospholipase C genes were evaluated by spotting conidia onto GFS medium containing 2.5 µM staurosporine (STS). The GFS medium contained 2% sorbose to produce compact colonies. (B) The levels of apoptosis in wild-type and Δ*plc-2* cells were detected by staining with YO-PRO1, and the percentage of positive cells was measured by flow cytometry. *P<0.05 (for 50 µM STS versus DMSO for each strain); ^#^*P*<0.05 (for STS-treated wild-type versus Δ*plc-2* cells). (C) [Ca^2+^]_c_ was measured in aequorin-expressing Δ*plc-2* cells after treatment with 20 µM STS. (D,E) Quantification (in arbitrary units, A.U.) of the [Ca^2+^]_c_ transients A and B, respectively, which are shown in C. Data show the mean±s.e.m.; *P<0.05. (F,G) Growth of wild-type and Δ*plc-2* strains in solid medium. The panels show growth at 48 h post-inoculation (F) and hyphal extension rates over time as the mean±s.e.m. (G). (H) Growth of wild-type and Δ*plc-2* strains in liquid medium over 24 hours was obtained by measuring absorbance (Abs) at 450 nm. Data show the mean±s.e.m. (I) Representative micrographs of wild-type and Δ*plc-2* strains at 8 hours and 24 hours after inoculation in liquid Vogel's minimal medium. The percentage of germinated cells is indicated in the upper right corner (mean±s.e.m.). Note the presence of swollen conidia and several ungerminated conidia in 24-hour-cultures of Δ*plc-2* (arrows). *P<0.05. Scale bars: 40 µm.

We examined the sensitivity to staurosporine of the deletion strains for the four predicted phospholipase C genes of *N. crassa* (Jung et al., 1997; Zelter et al., 2004). Interestingly, whereas Δ*plc-1* (ΔNCU06245) and Δ*plc-3* (ΔNCU09655) strains were slightly more resistant, Δ*plc-2* (ΔNCU01266) was substantially more resistant than the wild-type strain (Fig. 4A). We confirmed the increased resistance to staurosporine of Δ*plc-2* cells by measuring the levels of apoptosis with the YO-PRO1 marker (Idziorek et al., 1995). Treatment with staurosporine led to a significant increase in the percentage of apoptotic cells in the wild-type but not in the Δ*plc-2* deletion strain (Fig. 4B). A 2-hour treatment with staurosporine caused ∼23.4% apoptosis in wild-type and ∼9.9% in Δ*plc-2* cells. In the absence of PLC-2, the [Ca^2+^]_c_ response to staurosporine was nearly abolished altogether (Fig. 4C–E), paralleling the response in wild-type cells pre-treated with the phospholipase C inhibitor U-73122 (Fig. 3A). These results indicate that phospholipase C is required for staurosporine-induced cell death and support the conclusion that Ca^2+^ signaling is important during the process.

Radial growth of Δ*plc-2* cells in solid Vogel's minimal medium was severely delayed (Fig. 4F,G). Deletion of *plc-2* also resulted in very poor growth in liquid medium (Fig. 4H). After 8 hours of culture, almost all wild-type cells had germinated (95.8%) and normal hyphal elongation was observed (Fig. 4I). By contrast, Δ*plc-2* cells underwent greatly reduced germination (39.7% germination). The difference was also evident after 24 hours of growth; whereas the wild-type strain was fully developed with 99.9% germination and long branched and fused hyphae, the Δ*plc-2* knockout mutant only underwent 58.5% germination, possessed swollen cells and a few elongated branched hyphae with little cell fusion evident. The presence of some germinated hyphae in Δ*plc-2* knockout cultures might be explained by the redundant activity of the other phospholipase C genes, as suggested previously (Gavric et al., 2007).

### Staurosporine activates a putative TRP channel involving Ca^2+^ influx from the external medium

Because SOCE seems to be only partially involved in the staurosporine-induced Ca^2+^ signature (Fig. 3E), we further investigated the mechanism of Ca^2+^ influx responsible for the [Ca^2+^]_c_ peaks associated with phases A and B. So far, two Ca^2+^-uptake systems have been established in fungi – the high- and low-affinity Ca^2+^ uptake systems (HACS and LACS, respectively). At the molecular level, HACS comprises the CCH-1 and MID-1 channel (Muller et al., 2001) whereas the channel counterpart in LACS is FIG-1 (Muller et al., 2003).

**Fig. 5.**
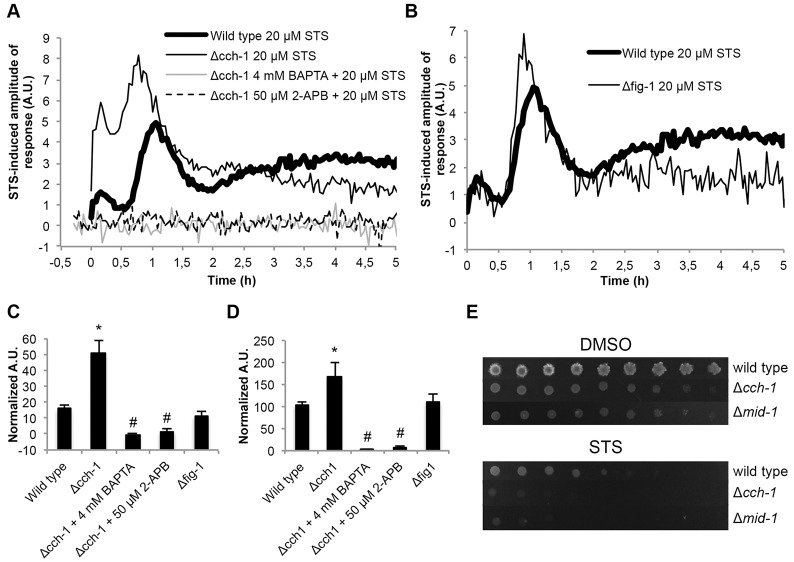
**Staurosporine-induced Ca^2+^ influx occurs through an uptake system distinct from the high- and low-affinity Ca^2+^ systems.** (A,B) The Ca^2+^ signature in response to 20 µM staurosporine (STS) was compared in aequorin-expressing wild-type and Δ*cch-1* (A) or Δ*fig-1* (B) cells. The influence of pre-treatment with 4 mM BAPTA and 50 µM 2-APB on the Δ*cch-1* STS-induced Ca^2+^ signature is shown in A. (C,D) Quantification (in arbitrary units, A.U.) of the [Ca^2+^]_c_ transients A and B, respectively, which are shown in panels A,B. Data show the mean±s.e.m.; *P<0.05 (for Δ*cch-1* versus wild-type cells); ^#^*P*<0.05 (for BAPTA and 2-APB pre-treated Δ*cch-1* cells versus Δ*cch-1* with STS alone). (E) The sensitivity of Δ*cch-1* and Δ*mid-1* strains was evaluated by spotting conidia onto GFS medium containing 2.5 µM STS.

A drastic increase in the [Ca^2+^]_c_ peaks associated with phases A and B was observed in aequorin-expressing Δ*cch-1* cells (ΔNCU02762) exposed to staurosporine (Fig. 5A,C,D). This was mainly due to extracellular Ca^2+^ uptake, because it was prevented by pre-treatment with the Ca^2+^-chelator BAPTA (Fig. 5A,C,D). The [Ca^2+^]_c_ response in the Δ*fig-1* mutant (ΔNCU02219) was more similar to that of the wild-type cells, despite a slight [Ca^2+^]_c_ amplitude increase in phase B and a decrease in phase C (Fig. 5B–D). These data strongly suggest that Ca^2+^ uptake during staurosporine-induced cell death involves channel activity distinct from that of the CCH-1–MID-1 high-affinity system or the FIG-1 low-affinity system. This unknown influx system was stimulated by staurosporine especially in the absence of CCH-1. Remarkably, complete abolition of the staurosporine [Ca^2+^]_c_ signature was observed when Δ*cch-1* cells were pre-treated with 2-APB (Fig. 5A,C,D), which is known to inhibit the IP_3_ receptor but also TRP channels (Clapham et al., 2005). As previously reported (Troppens et al., 2013), we could not generate a Δ*mid-1* strain expressing aequorin at sufficient levels for [Ca^2+^]_c_ measurement. MID-1 (NCU06703) is considered to be a regulatory partner of CCH-1 (Hong et al., 2013; Locke et al., 2000; Muller et al., 2001), although there are reports of MID-1 behaving as a non-selective stretch-activated cation channel protein (Cavinder et al., 2011; Kanzaki et al., 1999; Lew et al., 2008). Nonetheless, the knockout of *mid-1* phenocopied the *cch-1* deletion, as both strains showed defects such as reduced aerial hyphae, conidiation (supplementary material Fig. S3A) and mycelial extension rate (supplementary material Fig. S3B), consistent with both proteins acting together.

Given the accentuated Δ*cch-1* [Ca^2+^]_c_ signature during phases A and B in response to staurosporine (Fig. 5A), we assessed the susceptibility of Δ*cch-1* and Δ*mid-1* cells to staurosporine and observed that both strains are hypersensitive to the drug (Fig. 5E). This was corroborated by the analysis of the inhibitory effect of staurosporine after measuring both growth in liquid culture (supplementary material Fig. S3C) and YO-PRO1 staining to estimate apoptosis (supplementary material Fig. S3D). Deletion of *fig-1* resulted in a slight increase in susceptibility to staurosporine (supplementary material Fig. S4A). Thus, the upregulation of a Ca^2+^ influx system in the absence of CCH-1 (and possibly MID-1) is correlated with increased cell death.

**Fig. 6.**
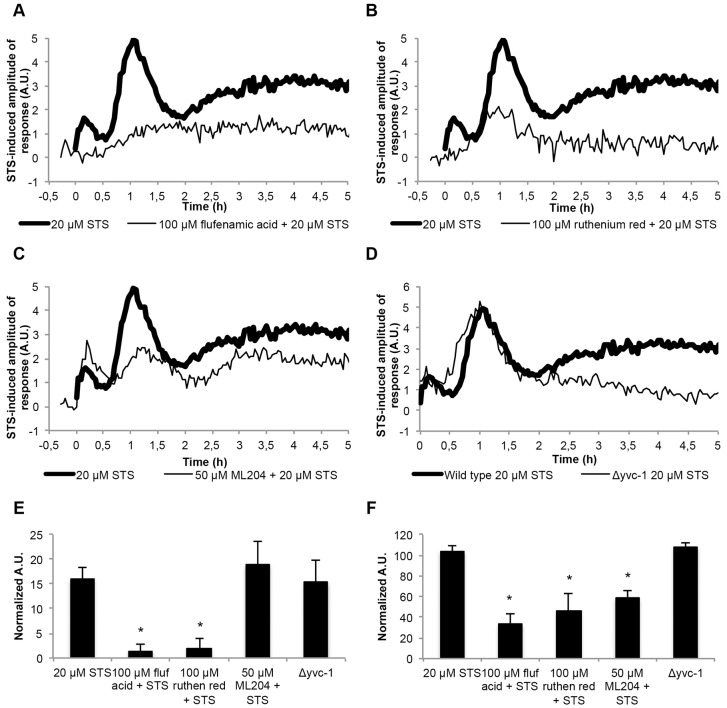
**Staurosporine activates a TRP-like channel responsible for extracellular Ca^2+^ influx.** (A–C) The Ca^2+^ signatures in response to 20 µM staurosporine (STS) in aequorin-expressing wild-type cells after pre-treatment with different Ca^2+^-modulating drugs. Cells were pre-treated with 100 µM flufenamic acid (A, fluf acid), 100 µM Ruthenium Red (B, ruthen red) and 50 µM ML204 (C). (D) The Ca^2+^ signatures in response to 20 µM STS in aequorin-expressing Δ*yvc-1* cells. (E,F) Quantification (in arbitrary units, A.U.) of the [Ca^2+^]_c_ transients A and B, respectively, which are shown in A–D. Data show the mean±s.e.m.; *P<0.05.

In animals, Ca^2+^ influx can be mediated by TRP channels, which are known to be regulated by the phospholipase C pathway (Rohacs, 2013). Because of the importance of phospholipase C during the response to staurosporine, we asked whether a TRP channel might be involved in the Ca^2+^ influx from the extracellular medium. The presence of flufenamic acid, a chemical that, albeit non-selectively, blocks some TRP channels (Guinamard et al., 2013), nearly abolished the staurosporine-induced [Ca^2+^]_c_ signature (Fig. 6A,E,F). We also performed [Ca^2+^]_c_ measurements in the presence of Ruthenium Red, a non-selective inhibitor of the MCU and a pan-inhibitor of TRP channels (Clapham et al., 2005), and found that the [Ca^2+^]_c_ response to staurosporine was also strongly suppressed (Fig. 6B,E,F). This is in agreement with our evidence that pre-incubation with 2-APB, an IP_3_-receptor inhibitor but also a blocker of TRP channels (Clapham et al., 2005), potently reduced the [Ca^2+^]_c_ response to staurosporine (Fig. 3D).

Because our data provides evidence that phospholipase C is activated by staurosporine and that the consequent depletion of PIP_2_ is an activation factor for a specific TRP channel, TRPC4 (Zhang et al., 2013), we analyzed the [Ca^2+^]_c_ response to staurosporine in the presence of ML204, a specific antagonist of TRPC4 and TRPC5 (Miller et al., 2011). With this inhibitor, the [Ca^2+^]_c_ peaks associated with phases B and C were significantly reduced (Fig. 6C,E,F). The effect of ML204 was not as drastic as that of the other TRP inhibitors, and this might be explained by the fact that it is very selective against mammalian TRPC4 and TRPC5 (Miller et al., 2011) and no obvious sequence homologs are present in *N. crassa*. Taken together, the differences in the [Ca^2+^]_c_ response to staurosporine-induced cell death in the presence of flufenamic acid, Ruthenium Red, 2-APB, ML204, BAPTA and U-73122, along with the deficient Ca^2+^ response of the Δ*plc-2* mutant strain, suggest that staurosporine induces the opening of a TRP-like channel regulated by phospholipase C.

So far, the only TRP channel reported in *N. crassa* has been YVC-1 (NCU07605 or NCU16725 in a recent annotation) (Zelter et al., 2004). The initial part of the staurosporine-induced [Ca^2+^]_c_ signature was similar in wild-type and Δ*yvc-1* cells (phases A and B), but [Ca^2+^]_c_ during phase C was decreased in Δ*yvc-1* cells (Fig. 6D–F). The growth of Δ*yvc-1* was inhibited by staurosporine, similar to that of the wild-type strain (supplementary material Fig. S4B). A role for this protein in extracellular Ca^2+^ uptake would not be expected, because the yeast homolog is localized to the vacuolar membrane and this channel mobilizes Ca^2+^ from the vacuole to the cytosol (Palmer et al., 2001). Thus, our combined pharmacological and genetic approach points to the existence of a Ca^2+^-permeable channel that has properties of a TRP-like channel, is distinct from YVC-1 and is the mediator of Ca^2+^ influx from the extracellular medium during staurosporine-induced cell death.

## DISCUSSION

The model organism *N. crassa* undergoes programmed cell death accompanied by the manifestation of several cellular phenotypes, such as DNA fragmentation, accumulation of reactive oxygen species (ROS), ceramide synthesis, compartmentalization of cells, glutathione export and activation of multidrug resistance proteins (Castro et al., 2010; Castro et al., 2008; Dementhon et al., 2006; Fernandes et al., 2013; Fernandes et al., 2011; Gonçalves and Videira, 2014; Plesofsky et al., 2008; Videira et al., 2009). Here, we show that staurosporine, an archetypal cell death inducer of therapeutic interest, activates a complex and dynamic intracellular response involving the influx of extracellular Ca^2+^ as well as the cytosolic recruitment of Ca^2+^ from intracellular stores. At the molecular level, this is promoted by a putative novel TRP-like channel and phospholipase C (probably PLC-2), which trigger a Ca^2+^ response with a characteristic [Ca^2+^]_c_ signature. Some reports have shown an increase in [Ca^2+^]_c_ in response to staurosporine (Dezaki et al., 2012; Himpens et al., 1993; Kruman et al., 1998; Norberg et al., 2008; Norberg et al., 2010; Seo and Seo, 2009), but the underlying mechanisms remain largely unknown. Recent research has shown that staurosporine, as well as its clinically relevant analog PKC412, induces Ca^2+^ influx through the hyperpolarization-activated cyclic nucleotide-gated channel HCN2 in human lung cancer cells and neurons (Norberg et al., 2010). No HCN2 homologs are known in *N. crassa*.

Our data indicate that staurosporine triggers the activation of phospholipase C by a currently unknown mechanism. Possible phospholipase C activation mechanisms include membrane stretch (Kinnunen, 2000), interaction with G-protein-coupled receptors (Werry et al., 2003) and liberation from phosphorylation by PKC (Ryu et al., 1990). Given that staurosporine is a powerful inhibitor of PKC (Omura et al., 1977), it is tempting to propose that it is the alleviation of the phosphorylation of phospholipase C by PKC that prompts the onset of the response. As a consequence of its activation, phospholipase C hydrolyses PIP_2_ with three outcomes – depletion of PIP_2_, generation of DAG and generation of IP_3_, all of which affect the activity of TRP channels (Rohacs, 2013). First, the depletion of PIP_2_ controls TRP proteins, although the mode of regulation depends on the specific identity of the channel; in animals, the depletion of PIP_2_ inhibits TRPC3, TRPC6 and TRPC7 (Lemonnier et al., 2008), whereas it activates TRPC4 (Zhang et al., 2013). Other channels, like TRPC5 and TRPV1 have been shown to be both negatively and positively regulated by PIP_2_ depletion, depending on the cellular environment (Rohacs, 2013). Our results support the hypothesis that phospholipase C depletes PIP_2_ and this causes the opening of a channel that behaves like TRPC4. Indeed, TRPC4 was shown to be inhibited by PKC (Venkatachalam et al., 2003), and we found that the selective TRPC4 antagonist ML204 partially reduced the [Ca^2+^]_c_ response to staurosporine. In the presence of staurosporine, PKC is inhibited (Omura et al., 1977), and this would relieve the inhibition of a TRPC4-like channel, allowing it to open. PIP_2_ depletion also affects TRPV1 (Chuang et al., 2001), but the existing data are conflicting on whether the reduction in PIP_2_ activates or inactivates the channel (Rohacs, 2013). Second, the generation of DAG might not have a significant role in our system, because DAG activates PKC, which is blocked by staurosporine alone (Omura et al., 1977). We cannot exclude that DAG is acting directly on TRP channels as shown previously (Hofmann et al., 1999; Venkatachalam et al., 2003). Nonetheless, it was shown that this PKC-independent direct interaction of DAG with TRP channels is protein specific and does not occur in members of the family such as TRPC4 and TRPC5 (Venkatachalam et al., 2003). If indeed there is a TRPC4-like channel involved in staurosporine-induced cell death, it is more likely that PIP_2_ depletion is the major regulatory mechanism. In accordance with our results, inositol starvation triggers ER-mediated cell death in fission yeast (Guérin et al., 2009). The generation of IP_3_ after treatment with staurosporine led to the mobilization of Ca^2+^ from internal stores, in line with the observation that during part of the response the ER contributes to the increase in [Ca^2+^]_c_.

The genome of *N. crassa* encompasses four putative phospholipase C (δ-type) genes (Jung et al., 1997; Zelter et al., 2004). Of the four, only PLC-1 was previously characterized, and it seems to be implicated in aspects of cell morphogenesis not involving polarized hyphal growth (Gavric et al., 2007). We observed that the absence of *plc-2* leads to aberrant spore germination and that it plays an important role in hyphal growth. Evidence has been previously presented from studies on *N. crassa* that support the idea that the IP_3_-mediated mobilization of Ca^2+^ from intracellular ER and Golgi-derived vesicles is involved in the maintenance of the tip-high Ca^2+^ gradient, which is reported to be required for hyphal elongation (Silverman-Gavrila and Lew, 2002). Our results suggest that PLC-2 might be the main phospholipase C engaged in this process. Recent observations in other filamentous fungi, however, have raised questions about the presence, and thus requirement, of a constant tip-high [Ca^2+^]_c_ gradient in continuously growing hyphae (Kim et al., 2012b). These authors have shown that transient [Ca^2+^]_c_ spikes occur in growing hyphal tips instead, but their role in hyphal elongation and the requirement for PLC-2 in generating these spikes is currently unclear. Other studies have shown that deletion of phospholipase C genes in *Magnaporthe oryzae* results in defects in other morphogenetic processes, such as appressorium formation and conidiation (Choi et al., 2011; Rho et al., 2009).

In *N. crassa*, treatment with the antifungal peptide PAF (Binder et al., 2010) or with the bacterial metabolite 2,4-diacetylphloroglucinol (DAPG) (Troppens et al., 2013) leads to Ca^2+^ influx from the external medium in a CCH-1-independent manner. DAPG-induced Ca^2+^ influx has also been shown to be independent of FIG-1, whereas this was not tested for PAF. Cell-survival-associated Ca^2+^ uptake, mediated by an unidentified channel, has been shown to occur in parallel with Ca^2+^ entry through Cch1–Mid1 in yeast treated with tunicamycin, an ER stress agent (Bonilla et al., 2002). There is thus evidence for the presence of fungal Ca^2+^ uptake systems in addition to the already characterized HACS (CCH-1 and MID-1) and LACS (FIG-1) mechanisms (Cavinder and Trail, 2012; Hong et al., 2013; Locke et al., 2000; Martin et al., 2011; Muller et al., 2001; Muller et al., 2003). Here, we describe a putative Ca^2+^ channel in the plasma membrane with the pharmacological properties of a TRP protein. This represents a novel mechanism for Ca^2+^ influx in fungi, and it is conceivable that it might be involved in some or all of these aforementioned responses. The only TRP channel so far described in *N. crassa* is YVC-1 (Zelter et al., 2004), although a few proteins were recently proposed as putative homologs of the mammalian TRP channels in pathogenic fungi (Prole and Taylor, 2012). It is not plausible that YVC-1 is the channel activated by staurosporine because the respective knockout mutant does not show major differences from wild-type cells in terms of the [Ca^2+^]_c_ response to staurosporine and, furthermore, yeast Yvc1 is localized in the vacuole (Palmer et al., 2001). The TRP Ca^2+^ uptake system seems to be more active in the absence of CCH-1. Possibly as a consequence of this upregulation in Ca^2+^ uptake, cells lacking CCH-1 (and MID-1) undergo more cell death than the wild-type cells when treated with staurosporine. This is consistent with the observation that Δ*cch-1* and Δ*mid-1* cells are less tolerant to high levels of Ca^2+^ (supplementary material Fig. S3E). In line with our results, lack of CCH-1 and MID-1 renders *S. cerevisiae* and *Cryptococcus neoformans* cells very sensitive to ER stress caused by tunicamycin or azole drugs (Bonilla et al., 2002; Hong et al., 2010; Martin et al., 2011).

**Fig. 7.**
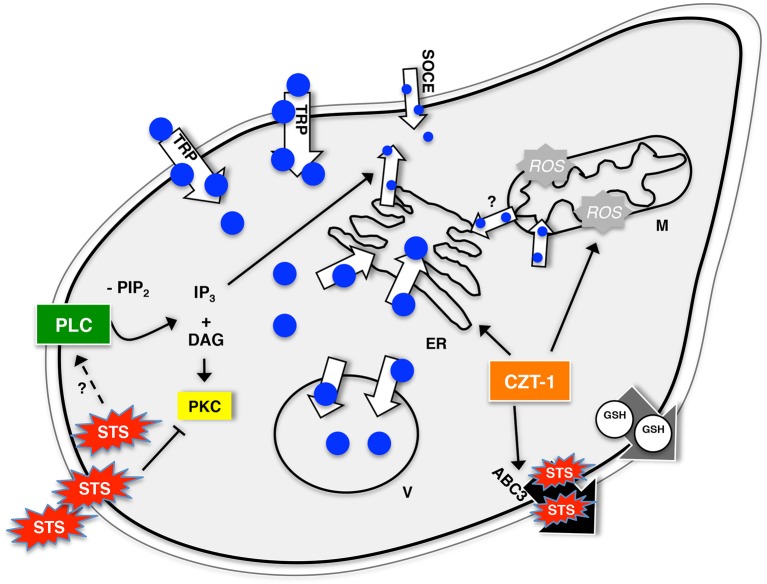
**A proposed model for the action of staurosporine in *N. crassa*.** See Discussion for details. Blue circles, Ca^2+^; M, mitochondria; ER, endoplasmic reticulum; V, vacuoles; ROS, reactive oxygen species; GSH, reduced glutathione; SOCE, store-operated Ca^2+^ entry; STS, staurosporine.

A model illustrating the mechanisms involved in staurosporine-induced and phospholipase-C-mediated cell death is presented in Fig. 7. We propose that the staurosporine-induced increase in the [Ca^2+^]_c_ is caused by continuous Ca^2+^ influx from the external medium (through an unidentified TRP-like channel and SOCE) and release of Ca^2+^ from internal stores by a variety of mechanisms. The action of staurosporine includes the activation of phospholipase C, which leads to the generation of IP_3_ and possibly to the regulation of the TRP-like channel. The vacuoles readily sequester the excess Ca^2+^ in an attempt to avoid the associated deleterious effects of Ca^2+^. This sequestration can occur by means of a Ca^2+^/H^+^ antiport system that is indirectly blocked by the disruptive effect of bafilomycin A1 on the proton gradient by inhibiting the vacuolar H^+^-ATPase (Cordeiro et al., 2011; Cordeiro et al., 2013). Sequestration of Ca^2+^ through this system seems to eventually saturate because bafilomycin A1 does not block the C phase of the staurosporine-induced Ca^2+^ signature. Interestingly, recent reports demonstrate that the vacuolar H^+^/ATPase is a central mediator of cell death in fungal (Kim et al., 2012a; Zhang et al., 2010) and cancer (von Schwarzenberg et al., 2013) cells. In *S. cerevisiae*, cells lacking vacuolar ATPase activity are very sensitive even to brief elevations in [Ca^2+^]_c_ (Forster and Kane, 2000). In future studies, it will be interesting to determine the role of the vacuolar H^+^-ATPase in *N. crassa* cell death.

Mitochondrial fluxes of Ca^2+^ also seem to be involved in the response to staurosporine. The initial changes in the staurosporine-induced Ca^2+^ signature when the MCU is inhibited with Ru360 suggest that mitochondria play an early role in Ca^2+^ sequestration. However, [Ca^2+^]_c_ peaks B and C are reduced in the presence of Ru360, suggesting that the role of mitochondria is not restricted to Ca^2+^ sequestration but also involves either direct release of Ca^2+^ into the cytosol or the ER through the so-called microdomains of contact between the two organelles (Clapham, 2007). Interestingly, thapsigargin-treated cells generated the opposite response to those treated with Ru360, because the amplitudes of the [Ca^2+^]_c_ transients increased during phases B and C. These results can be possibly explained by the hypothesis that mitochondria and ER exhibit Ca^2+^ crosstalk over long periods by transporting Ca^2+^ ions between them. The apparent paradoxical conclusion that the ER is releasing Ca^2+^ through an IP_3_-activated channel but sequestering the ion at the same time (especially during phase B, see the staurosporine-induced cytosolic [Ca^2+^]_c_ transient in thapsigargin-treated cells, Fig. 2B) might be explained by a tunneling mechanism by which the ER can load with Ca^2+^ through contact spots between mitochondria and ER, and almost instantaneously release Ca^2+^ from regions of the ER that are rich in IP_3_-activated channels (Petersen and Verkhratsky, 2007). Extracellular Ca^2+^ uptake continues throughout the whole duration of the response to staurosporine, as deduced from the experiments involving treatment with the Ca^2+^-chelator BAPTA. Our model also illustrates that the extent of staurosporine-induced cell death is determined by the activity of the staurosporine-exporting ATP-binding cassette transporter ABC3 (Fernandes et al., 2011), which is under the control of the transcription factor CZT-1 (Goncalves et al., 2014). ROS accumulation is also controlled by CZT-1 (Goncalves et al., 2014) and is required for staurosporine-induced cell death, which, in turn, is facilitated by the efflux of reduced glutathione (GSH) (Castro et al., 2010; Fernandes et al., 2013).

This study has made extensive use of inhibitors that are commonly used to experimentally manipulate Ca^2+^ signaling in mammalian cells, and some of these drugs lack specificity against their purported targets. Therefore, some caution needs to be taken with regard to the interpretation of results from the inhibitor experiments, especially because the precise targets of these inhibitors in fungal cells have not been characterized in detail. Of the inhibitors employed in our study, several have been previously used in *N. crassa* – thapsigargin (Hamam and Lew, 2012), bafilomycin A1 (Bowman et al., 2004) and U-73122 (Silverman-Gavrila and Lew, 2002). Ru360, xestospongin C, YM-58483 and ML204 have not been used previously with *N. crassa* cells but are known to be very selective in animal cells (Gafni et al., 1997; Ishikawa et al., 2003; Matlib et al., 1998; Miller et al., 2011). Despite these limitations, our pharmacological results are consistent with those obtained using a genetic approach.

Staurosporine is a widely used tool in cell death research and a prototype for anticancer drugs (Gani and Engh, 2010). Thus, characterization of its mechanism of action might impact on future studies on the fundamental aspects of signaling during cell death and on the development of therapeutic treatments for fungal infection and cancer.

## MATERIALS AND METHODS

### Strains, culture media and chemicals

*N. crassa* was handled according to standard procedures. Vogel's minimal medium plus 1.5% (w/v) sucrose was used in all experiments (Davis and de Serres, 1970). Wild-type and deletion strains used in this study are listed in supplementary material Table S1. The following chemicals were used: staurosporine (LC Laboratories, Woburn, MA); DMSO, ML204, flufenamic acid and xestospongin C (Sigma-Aldrich, St Louis, MO); 4-methyl-4′-[3,5-bis(trifluoromethyl)-1H-pyrazol-1-yl]-1,2,3-thiadiazole-5-carboxanilide (YM-58483/BTP2) and 2-aminoethoxydiphenyl borate (2-APB) (Tocris Bioscience, Bristol, UK); 1,2-bis(ortho-aminophenoxy)ethane-N,N,N′,N′-tetrasodium (BAPTA), thapsigargin, Ru360, LiCl_2_ and 7-hydroxystaurosporine (UCN-01) (Merck Millipore, Darmstadt, Germany); 1-[6-((17β-3-methoxyestra-1,3,5(10)-trien-17-yl)amino)hexyl]-1H-pyrrole-2,5-dione (U-73122) (Alexis Biochemicals, San Diego, CA) and bafilomycin A1 (Wako Chemicals, Richmond, VA).

Mycelial extension rates at 26°C were measured after inoculating 5 µl containing 1×10^3^ conidia onto the centre of large Petri dishes (14.2-cm diameter) containing solid minimal medium. For growth measurements in liquid minimal medium, 1×10^4^ conidia/ml were incubated at 26°C, 100 rpm, under constant light in 96-well plates (200 µl total volume per well) and absorbance was monitored at 450 nm over 24 hours.

### Intracellular Ca^2+^ measurement with aequorin

Genetically encoded Ca^2+^ reporters are the most reliable option for the study of intracellular Ca^2+^ dynamics, and such a method was developed for filamentous fungi using the bioluminescent Ca^2+^ reporter aequorin that has been codon-optimized for *N. crassa* (Nelson et al., 2004). The pAB19 vector containing the gene encoding the synthetic aequorin was used to transform *N. crassa* cells by electroporation with an Eppendorf Multiporator (Hamburg, Germany) at 1800 V for 5 ms. Aequorin-expressing conidia were obtained and incubated at a concentration of 2×10^6^ cells/ml with 5 µM coelenterazine (Santa Cruz Biotechnology, Dallas, TX) in minimal medium. Aliquots of 100 µl were added to each well of white opaque 96-well plates and incubated for 6 hours at 26°C in the dark without agitation. The luminescence emitted was measured in relative light units (RLU) using a Bio-Tek Synergy HT (Winooski, VT) microplate reader. Owing to equipment constraints, it was not possible to convert RLU values into precise [Ca^2+^]_c_ concentrations. Therefore, the luminescence results were normalized for each strain to allow direct comparisons between different experiments as follows. For each strain, 100 µl of 3 M CaCl_2_ in 20% ethanol was pipetted into extra wells of the 96-well plates and discharged aequorin luminescence was measured for 3 minutes. This provided a measurement of the total aequorin luminescence that could be emitted and corresponds to the maximum level of aequorin expressed for each strain in each experiment. These total aequorin discharge measurements were used to normalize the experimental RLU values for each strain [cytosolic Ca^2+^ levels (arbitrary units) = experimental RLU values/total emitted luminescence]. For each plot of the values of aequorin luminescence, as a readout of [Ca^2+^]_c_, quantification was performed by summing the normalized experimental values, and data are expressed as the mean±s.e.m. The RLU values of solvent DMSO control samples were subtracted from the RLU values of the staurosporine-treated samples to obtain the ‘staurosporine-induced Ca^2+^ amplitude of response’. Where specified, samples were pre-incubated for 15 minutes with various pharmacological agents before staurosporine (or DMSO) was added. In all instances, the volume of chemical(s) added to the wells was 10 µl (from an appropriate stock solution), to ensure good homogenization. The aequorin luminescence plots presented in this paper correspond to the average of at least three independent experiments with three to six replicates per experiment. The typical staurosporine-induced Ca^2+^ signature of wild-type cells was obtained from 30 independent experiments.

### Cell death assays

For the spot assay, nine successive threefold dilutions were prepared for each strain starting with 6.6×10^7^ cells/ml. From each dilution, 5 µl was spotted onto plates containing glucose-fructose-sorbose (GFS) medium with agar supplemented with the indicated chemical. Cells were incubated at 26°C and images were obtained 72 hours later.

For the detection of apoptotic cells by flow cytometry, the fluorophore YO-PRO1 (Life Technologies, Carlsbad, CA) was used. Conidia at a concentration of 10^6^ cells/ml were cultured for 4 hours in Vogel's minimal medium at 26°C, 140 rpm, under constant light conditions, followed by the addition of staurosporine and growth for a further 2 hours. Samples were harvested by centrifugation, washed twice with PBS and incubated with 0.1 µM YO-PRO 1. After 20 minutes on ice, samples were read in a Beckman-Coulter EPICS XL-MCL (Brea, CA). Results represent the mean±s.e.m.

### Microscopy

For routine microscopy, a Nikon TE2000E inverted microscope with a 60×/1.2 NA water-immersion plan apo objective (Nikon, Kingston-upon-Thames, UK) and differential interference contrast (DIC) optics was used. For these analyses, 200-µl drops of conidial suspension in liquid Vogel's medium containing 5×10^5^ conidia/ml were placed in eight-well slide culture chambers (Nalg Nunc, Rochester, NY) and incubated at 26°C. Images were captured with an ORCA-ER CCD camera (Hamamatsu, Welwyn Garden City, UK) driven by the MetaMorph NX1.1 software for image acquisition. The percentage of germinated cells was calculated using ImageJ (NIH, Bethesda, MD).

For the evaluation of propidium iodide uptake by dead cells, conidia at a concentration of 2×10^6^ cells/ml were incubated in eight-well slide chambers at 26°C in the dark without agitation and stained with 5 µg/ml propidium iodide (Sigma-Aldrich). After 6 hours of growth, 20 µM staurosporine or DMSO was applied and images were obtained at appropriate time-points. Micrographs were obtained using an Olympus IX81 inverted fluorescence microscope (Tokyo, Japan) equipped with DIC optics. Fluorescence images were acquired using a plan fluor 20×/0.45 NA objective lens, using a 100-W mercury fluorescence light source and a BP 510–550 excitation filter; fluorescence emission was filtered through a dichroic mirror DM570 (Olympus, Tokyo, Japan). Images were acquired with a cooled CCD camera (ColorView II, Soft Imaging System GmbH, Münster, Germany) connected to a computer running Cell F (Olympus, Tokyo, Japan). The exposure time was adjusted to 50 ms without binning. The percentage of propidium-iodide-positive cells was quantified using ImageJ (NIH) and expressed as the mean±s.e.m.

### Statistical analysis

Statistical analysis of the data was performed using SPSS 20 (SPSS, Chicago, IL). The non-parametric Mann–Whitney test was used for comparisons between two groups. *P*-values of ≤0.05 were considered statistically significant.

## Supplementary Material

Supplementary Material
