# Supplementary material for: Activation of a TRP-like channel and intracellular Ca2+ dynamics during phospholipase-C-mediated cell death
Source: J Cell Sci. 2014 Sep 1;127(17):3817–29. doi: 10.1242/jcs.152058 (PMC4150065; doi:10.1242/jcs.152058)
Supplement: Supplementary Material [file supp_127_17_3817__index.html]

Activation of a TRP-like channel and intracellular Ca2+ dynamics during phospholipase-C-mediated cell death — Supplementary Material 

# Activation of a TRP-like channel and intracellular Ca2+ dynamics during phospholipase-C-mediated cell death

## JCS152058 Supplementary Material

**Files in this Data Supplement:**

- **Supplementary Material**
