# Supplementary material for: Activation of a TRP-like channel and intracellular Ca2+ dynamics during phospholipase-C-mediated cell death
Source: J Cell Sci. 2014 Sep 1;127(17):3817–29. doi: 10.1242/jcs.152058 (PMC4150065; doi:10.1242/jcs.152058)
Supplement: Supplementary Material [file supp_127.17.3817_JCS152058.pdf]

Activation of a TRP-like channel and intracellular calcium dynamics during phospholipase C-mediated cell death

A. Pedro Gonçalves [1,2,a,b], J. Miguel Cordeiro [2,a], João Monteiro [1], Alberto Muñoz [3], Paulo Correia-de-Sá [2], Nick D. Read [3], Arnaldo Videira [1,2,b]

(1) IBMC-Instituto de Biologia Molecular e Celular - Universidade do Porto, Rua do Campo Alegre 823, 4150-180 Porto, Portugal

(2) ICBAS-Instituto de Ciências Biomédicas de Abel Salazar, Universidade do Porto, Rua de Jorge Viterbo Ferreira 228, 4050-313 Porto, Portugal

(3) Manchester Fungal Infection Group, Institute of Inflammation and Repair, CTF Building, Grafton Street, University of Manchester, Manchester M13 9NT, UK

[a] These authors contributed equally to this work.

[b] To whom correspondence should be addressed: apgoncalves@ibmc.up.pt; avideira@ibmc.up.pt

Supplemental Figures

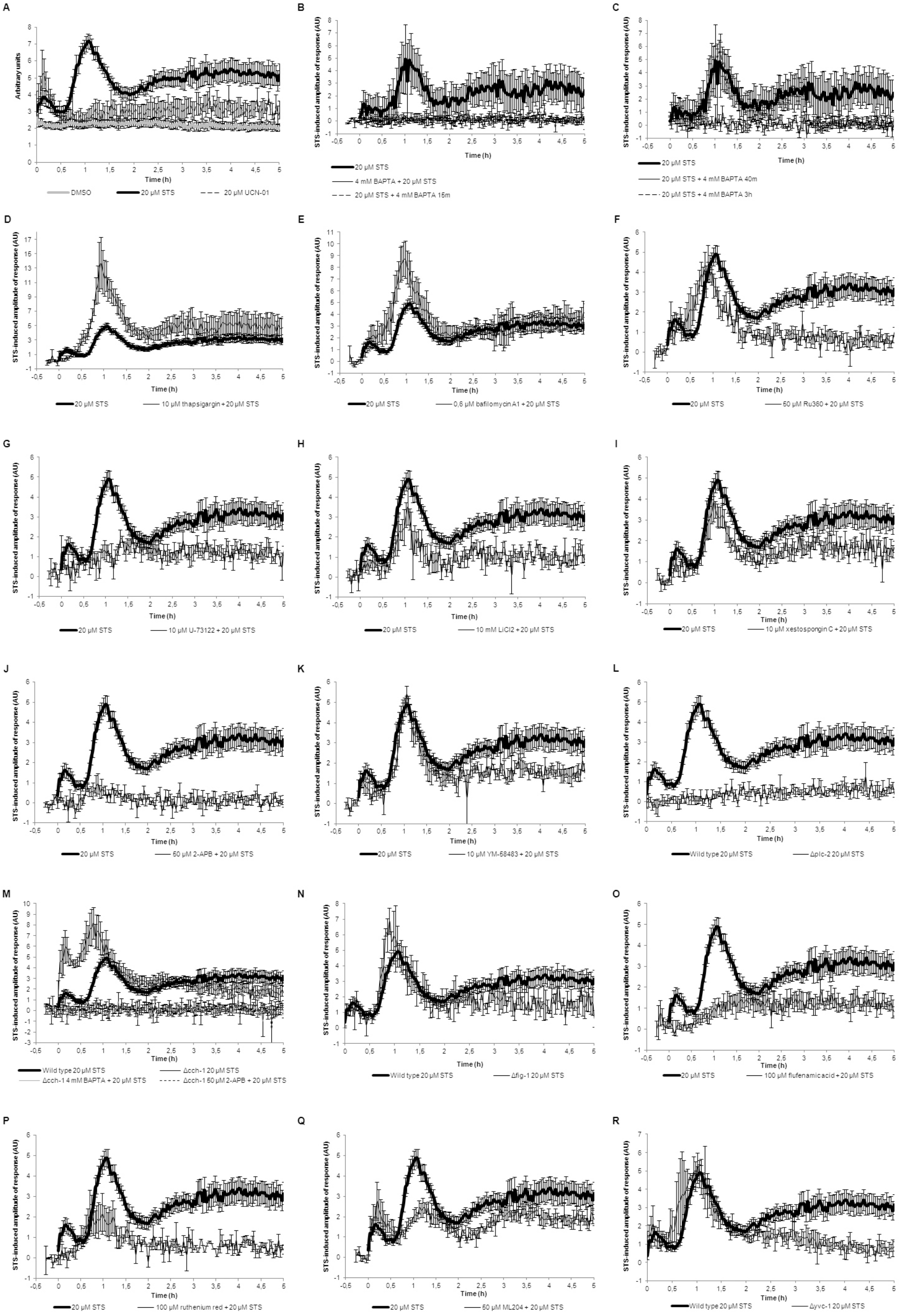
A
Arbitrary units
Time (h)
DMSO
20 µM STS
20 µM UCN-01
B
STS-induced amplitude of response (AU)
Time (h)
20 µM STS
4 mM BAPTA + 20 µM STS
20 µM STS + 4 mM BAPTA 15m
C
20 µM STS
20 µM STS + 4 mM BAPTA 40m
20 µM STS + 4 mM BAPTA 3h
D
10 µM thapsigargin + 20 µM STS
E
0,6 µM bafilomycin A1 + 20 µM STS
F
50 µM Ru360 + 20 µM STS
G
10 µM U-73122 + 20 µM STS
H
10 mM LiCl2 + 20 µM STS
I
10 µM xestospongin C + 20 µM STS
J
50 µM 2-APB + 20 µM STS
K
10 µM YM-58483 + 20 µM STS
L
Wild type 20 µM STS
Δplc-2 20 µM STS
M
Δcch-1 20 µM STS
Δcch-1 4 mM BAPTA + 20 µM STS
Δcch-1 50 µM 2-APB + 20 µM STS
N
Δfig-1 20 µM STS
O
100 µM flufenamic acid + 20 µM STS
P
100 µM ruthenium red + 20 µM STS
Q
50 µM ML204 + 20 µM STS
R
Δyvc-1 20 µM STS

Supplemental Figure 1 - The staurosporine-induced cytosolic Ca^{2+} signature and its modulation by different inhibitors or by deletion of specific genes. The curves are the same as shown in Figures 1-6, but include error bars (s.e.m.).

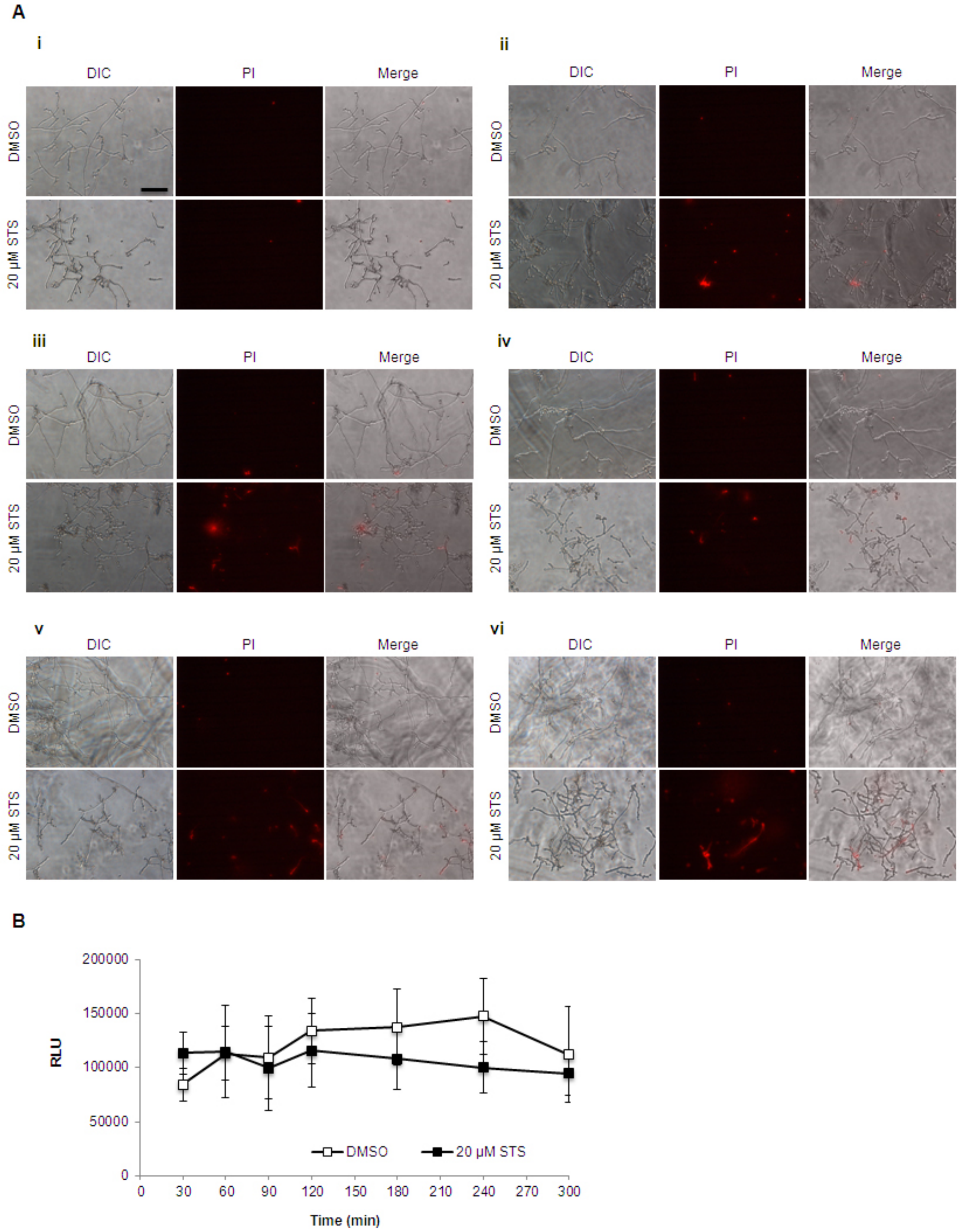

Supplemental Figure 2 - Staurosporine-induced cell death kinetics. (A) Cell death was measured in staurosporine-treated cells after propidium iodide (PI) staining in 6-hour cultures.

Representative micrographs at the 45 (i), 90 (ii), 135 (iii), 180 (iv), 240 (v) and 300 (vi) minutes time points after the addition of staurosporine (STS) or DMSO. Scale bar: 30 µm. (B) Total available aequorin present in aequorin-expressing wild type cells grown for 6 hours and incubated with 20 µM staurosporine (STS) or DMSO (untreated control). The curves show measurements of relative light units (RLU) at the indicated time points after the injection of 100 µl of 3M $CaCl_2$ in 20% ethanol to complete discharge the aequorin.

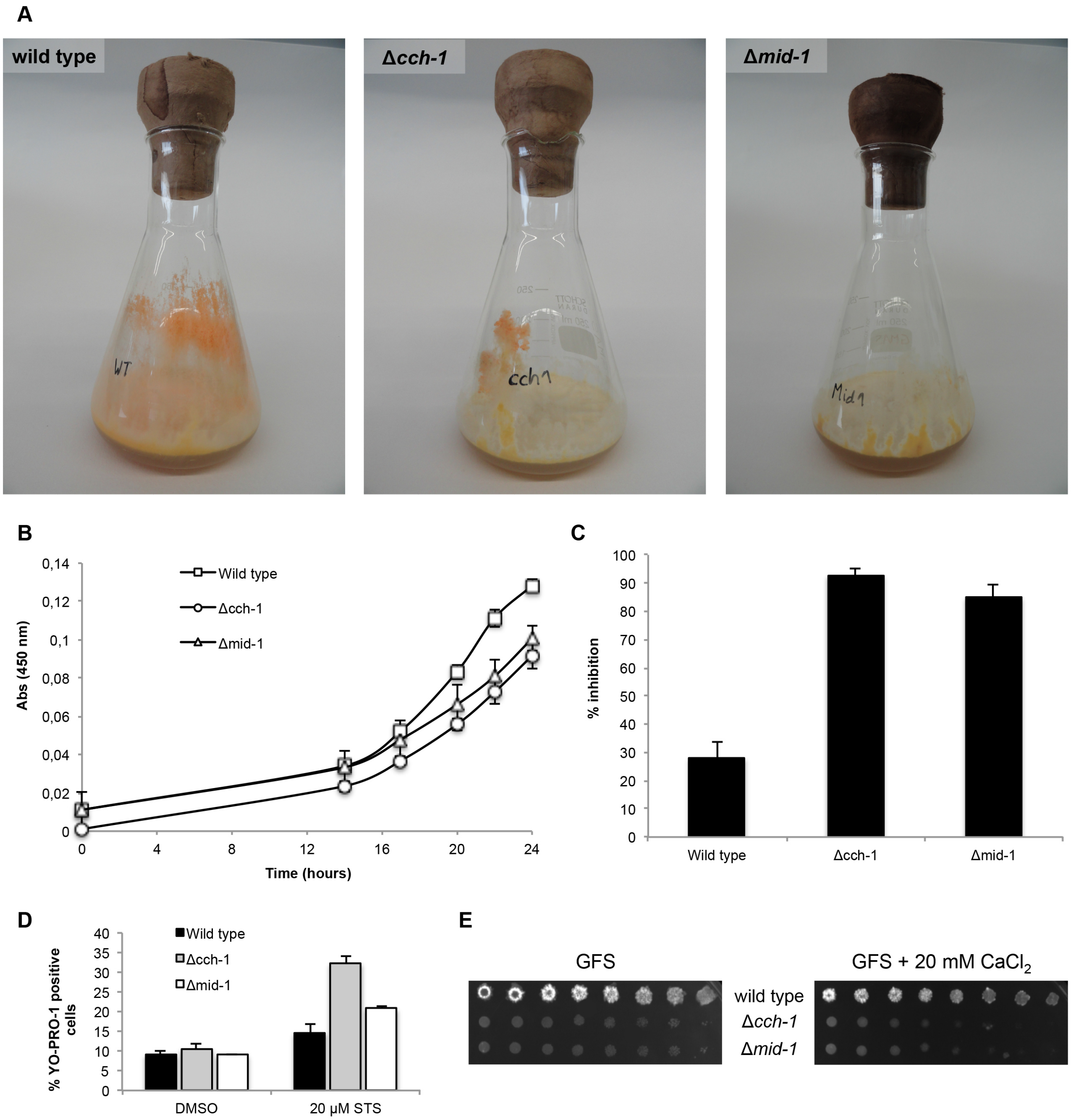

Supplemental Figure 3 - Deletion of *cch-1* and *mid-1* results in abnormal development of aerial hyphae, conidiation, growth rate and an increased susceptibility to staurosporine and $CaCl_2$. (A) Wild type, Δ*cch-1* and Δ*mid-1* cells grown in solid Vogel's minimal medium for 7 days. (B) Growth of wild type, Δ*cch-1* and Δ*mid-1* cells over 24 hours by measuring absorbance at 450 nm. (C) Percentage of growth inhibition caused by a 24-hour treatment with 2.5 μM staurosporine, as determined by measuring absorbance at 450 nm. (D) The levels of staurosporine-induced apoptosis in wild type, Δ*cch-1* and Δ*mid-1* detected by staining with YO-

PRO1 and determining the percentage of positive cells by flow cytometry. (E) The growth of wild type, Δ*cch-1* and Δ*mid-1* in the presence of 20 mM $CaCl_2$ evaluated by the spot assay.

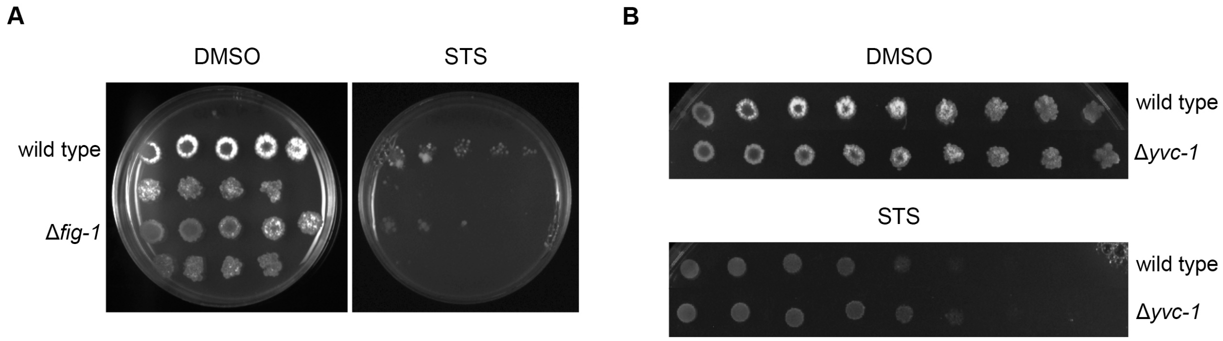

Supplemental Figure 4 - Staurosporine sensitivity profile of (A) Δ*fig-1* and (B) Δ*yvc-1* mutant strains, evaluated by spotting conidia on GFS medium containing 2.5 μM staurosporine (STS).

Table S1. ***N. crassa*** **strains used in this study**

Strain	Genotype	Source, References
Wild type	wt *mat A*	FGSC #2489
Wild type aequorin	*mat A*, *hygR*, *aeqS*	(Nelson et al., 2004)
Δ*plc-1*	ΔNCU06245, *mat a*, *hygR*	FGSC #11411
Δ*plc-2*	ΔNCU01266, *mat a*, *hygR*	FGSC #12022
Δ*plc-2* aequorin	ΔNCU01266, *mat a*, *hygR*, *bar*, *aeqS*	This study
Δ*plc-3*	ΔNCU09655, *mat a*, *hygR*	FGSC #11271
Δ*plc-4*	ΔNCU02175, *mat a*, *hygR*	FGSC #12023
Δ*cch-1*	ΔNCU02762, *mat A*, *hygR*	(Troppens et al., 2013)
Δ*cch-1* aequorin	ΔNCU02762, *mat A*, *hygR*, *bar*, *aeqS*	(Troppens et al., 2013)
Δ*mid-1*	ΔNCU06703, *mat a*, *hygR*	FGSC #11707
Δ*fig-1*	ΔNCU02219, *mat a*, *hygR*	FGSC #17273
Δ*fig-1* aequorin	ΔNCU02219, *mat a*, *hygR*, *bar*, *aeqS*	This study
Δ*yvc-1*	ΔNCU07605, *mat A*, *hygR*	FGSC #11253
Δ*yvc-1* aequorin	ΔNCU07605, mat A, *hygR*, *bar*, *aeqS*	(Troppens et al., 2013)

FGSC, Fungal Genetics Stock Center (McCluskey et al., 2010).
